# Use of the CytoSorb adsorber in patients with acute-on-chronic liver failure

**DOI:** 10.1038/s41598-024-61658-3

**Published:** 2024-05-17

**Authors:** Patrick Haselwanter, Bernhard Scheiner, Lorenz Balcar, Georg Semmler, Marlene Riedl-Wewalka, Monika Schmid, Thomas Reiberger, Christian Zauner, Mathias Schneeweiss-Gleixner

**Affiliations:** Intensive Care Unit 13H1, Division of Gastroenterology and Hepatology, Department of Medicine III, Medical University of Vienna, Waehringer Guertel 18-20, 1090 Vienna, Austria

**Keywords:** Hepatology, Alcoholic liver disease, Liver cirrhosis

## Abstract

CytoSorb is a hemoadsorptive column used to remove high concentrations of proinflammatory cytokines in septic shock. Data on CytoSorb application in acute-on-chronic liver failure (ACLF) is lacking. This retrospective observational study analyzed 21 ACLF patients admitted to ICUs at the Vienna General Hospital who received CytoSorb adsorber therapy between 2017 and 2023. Median ICU length of stay was 8 days (IQR: 3–13), the ICU survival rate was 23.8% (n = 5). Significant decreases in bilirubin (median peak: 20.7 mg/dL to median post-treatment: 10.8 mg/dL; − 47.8%; *p* < 0.001), procalcitonin (1.34 to 0.74 pg/mL; − 44.6%; *p* < 0.001), interleukin-6 (385 to 131 ng/mL; − 66.0%; *p* = 0.0182)—but also of platelets (72 to 31 G/L; − 56.9%; *p* = 0.0014) and fibrinogen (230 to 154 mg/dL; − 33.0%; *p* = 0.0297) were detected. ICU survivors had a trend towards a stronger relative decrease in bilirubin (− 76.1% vs. − 48.2%), procalcitonin (− 90.6% vs. − 23.5%), and IL-6 (− 54.6% vs. − 17.8%) upon CytoSorb treatment. Moreover, no serious CytoSorb-attributed complications were detected. In conclusion, use of CytoSorb adsorber in ACLF patients results in a significant decrease in bilirubin and proinflammatory cytokines, while platelets and fibrinogen were also lowered. Prospective trials are warranted to investigate the impact of CytoSorb on clinical outcomes of ACLF patients with high proinflammatory cytokine levels.

## Introduction

Acute-on-chronic liver failure (ACLF) is a syndrome that may occur in patients with cirrhosis experiencing acute decompensation and is characterized by the development of hepatic and/or extra-hepatic organ failure(s), resulting in excessive short-term mortality^1^. It has been demonstrated that systemic inflammation is the hallmark of ACLF driving disease development and progression^1–8^.

The underlying mechanisms leading to systemic inflammation in ACLF are only partly understood and depend on the specific precipitating event, which may be identified in up to 60% of cases^7^. Inflammation is associated with the release of damage-associated (DAMPs) or pathogen-associated molecular patterns (PAMPs), resulting in oxidative stress triggering immune-mediated tissue damage, mitochondrial dysfunction, renal hypoperfusion, and subsequently, single or multiple organ failures^3,9^. In line, Monteiro and colleagues recently demonstrated the impact of the inflammasome, as assessed by the pro-inflammatory cytokines interleukin (IL)-1α and IL-1β, on ACLF outcome^10^. Therefore, amelioration of systemic inflammation, e.g., by clearance of pro-inflammatory cytokines, may be an important therapeutic goal in ACLF patients in order to promote liver regeneration or as a bridging therapy to liver transplantation^5,11,12^.

The CytoSorb adsorber is an extracorporeal blood purification tool (hemoadsorber) that may be used together with continuous dialysis to remove excessive inflammatory mediators (i.e., cytokines), as well as to lower elevated bilirubin and myoglobin levels^13^. Regarding liver-related indications, there is some data on the use of CytoSorb in patients with isolated hyperbilirubinemia and case series on liver failure^14^. However, there is no data on the use of CytoSorb in the specific setting of ACLF. Previously, extracorporeal liver support devices failed to improve the prognosis of patients with ACLF^15^. Extracorporeal treatment with large-volume plasma exchange—in order to eliminate pro-inflammatory cytokines—is explored as a promising treatment option in ACLF^16^. Moreover, a recently published randomized, controlled clinical trial detected a significant decrease in biomarkers involved in the pathophysiological process of ACLF in patients treated with the liver dialysis system DIALIVE compared to patients with standard medical treatment. Patients treated with the DIALIVE device showed an improvement in CLIF-C OF and CLIF-C ACLF scores but no significant difference in 28-day mortality^17^. The results of a phase III study investigating the efficacy of plasma exchange in ACLF patients are still pending (APACHE, NCT03702920)^18^.

Given the unmet clinical need for supportive treatments in patients with ACLF, we aimed to investigate the impact of CytoSorb treatment on liver and extra-hepatic organ function and to evaluate potential complications occurring during this treatment in critically ill ACLF patients.

## Methods

### Study design and setting

We conducted a retrospective observational study of patients with ACLF admitted to the ICU at a large tertiary center (Vienna General Hospital). Importantly, we did not include patients with acute liver failure (ALF) without pre-existing liver disease. All adult (18 years and older) patients with ACLF receiving treatment with CytoSorb adsorber between January 1st, 2017, and January 1st, 2023, were included in this study. The diagnosis of ACLF was defined according to the European Association for the Study of the Liver (EASL) CLIF criteria^19^. The observation period started from ICU admission (baseline) until ICU discharge or death. We evaluated the effects of CytoSorb hemoadsorption on the clinical course, carefully screened for potential complications, and analyzed laboratory parameters directly prior to CytoSorb application, after 24 h, and at the end of CytoSorb treatment (or last available in case of death).

In addition, we included a control group comprising 10 patients with ACLF who were all treated at the ICU during the respective time period 2017 to 2023 and received hemodialysis without the inclusion of the CytoSorb adsorber.

### Hemoadsorption with CytoSorb adsorber

All included ACLF patients fulfilled an indication (i.e., acute kidney injury, hyperammonemia, and fluid- and acid–base disturbances) for hemodialysis during their respective ICU stay. The decision for additional CytoSorb adsorber application was made by the treating physicians. Thereby, CytoSorb adsorber was always used in addition to standard intensive care treatment of ACLF patients according to current EASL guidelines^19^. CytoSorb adsorber was always used with continuous venovenous hemodialysis (CVVHD; MultiFiltrate, Fresenius Medical Care) in prefilter position and changed after 8–24 h. Anticoagulation during extracorporeal blood circulation was primarily conducted with citrate. In case of suspected citrate accumulation, the anticoagulation regimen was switched to antithrombin III supplementation. According to our local standard operating procedures, citrate accumulation is rigorously monitored at least 3 times daily in patients with liver failure using the total calcium to ionized calcium ratio^20^. Dialysis with low-molecular-weight heparin was not applied in our patient cohort, nor was dialysis without anticoagulation^21^.

Laboratory parameters including liver chemistry [bilirubin, aspartate transferase (AST), alanine aminotransferase (ALT), alkaline phosphatase (aP), gamma-glutamyl transferase (GGT), and ammonia], inflammation parameters [procalcitonin (PCT), interleukin-6 (IL-6), C-reactive protein (CRP)], coagulation parameters [fibrinogen, international normalized ratio (INR)], white blood count (WBC) and platelets were collected in all patients and analyzed prior to CytoSorb application, 24 h after the commencement of CytoSorb therapy and after discontinuation of CytoSorb therapy (or prior to death). Overall CytoSorb treatment time and all CytoSorb adsorber changes were documented. For the evaluation of potential adverse events or complications directly associated with CytoSorb therapy, the patient's condition and laboratory changes were closely monitored during hemoadsorption in standardized time intervals.

### Data collection

Data were extracted from electronic patient charts (IntelliSpace Critical Care and Anesthesia, Philips, Amsterdam, Netherlands) that are routinely used at all ICUs at the Vienna General Hospital. The system enables prospective and digital documentation of crucial patient data, including patient characteristics (age, gender, height, weight, BMI, vital signs and comorbidities/underlying disease), laboratory tests (blood chemistry, global tests of coagulation and blood cell count) and complete information about ICU-specific parameters like fluid balances, medication, nutrition and extracorporeal life support (mechanical ventilation, renal replacement therapy and ECMO). In order to quantify the severity of critical illness and extent of organ dysfunction for patients admitted to the ICU, we calculated the following scores within the first 24 h upon ICU admission: simplified acute physiology score (SAPS II)^22^ and sequential organ failure assessment score (SOFA)^23^. In addition, CLIF-C-ACLF and CLIF-C-OF scores were calculated as ACLF-specific prognostic scores^24,25^. Child–Pugh-Score (CPS) was collected using laboratory data and clinical parameters at ICU admission^26,27^.

### Statistical analysis

Descriptive statistical analysis was used to provide a demographic overview of our patient cohort. Continuous variables were reported as mean ± standard deviation or median (interquartile range), while categorical variables were reported as numbers (relative proportions, %). Differences in laboratory parameters before and after CytoSorb adsorber application were compared using the Wilcoxon test for non-parametric variables. All statistical analyses were performed using IBM SPSS Statistics 27 (IBM, New York, NY, USA) and GraphPad Prism 8 (GraphPad Software, CA, USA).

### Ethical approval and informed consent

The study was conducted according to the guidelines of the Declaration of Helsinki^28^, and approved by the local Ethics Committee of the Medical University of Vienna (Ethics committee number: 1924/2020). Given the retrospective design of the study, informed consent requirement was waived by the ethics committee of the Medical University of Vienna.

## Results

### Patient characteristics

**Figure 1:**
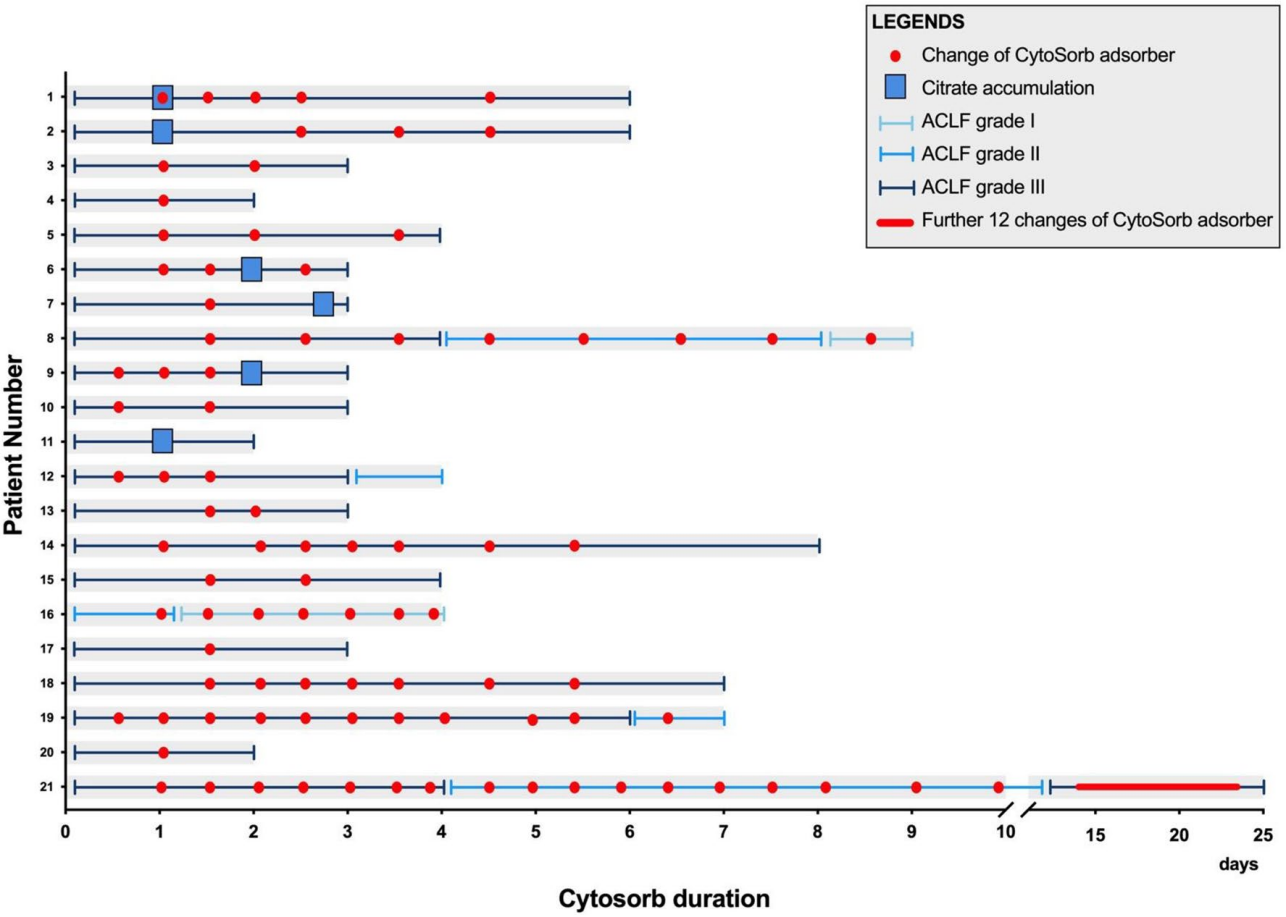
Individual patients' courses during CytoSorb adsorber application. The patient numbers refer to Table 2. CytoSorb adsorber changes were highlighted with red dots. The evolution of ACLF Grade is represented by bars of different colors as indicated.

During the study period, 21 patients with ACLF were admitted to the ICU and received CytoSorb adsorber therapy (Supplemental Figure S1). The baseline characteristics of our patient population are depicted in Table 1. The individual characteristics of each patient are shown in Table 2. The median age was 50 years (IQR: 35–58), and most patients were male (n = 18; 86%). SOFA and SAPS II scores at ICU admission were 16 (IQR: 13–19) and 59 (IQR: 53–69), respectively. The most common underlying etiology of cirrhosis was alcohol-related liver disease (ALD; n = 11), followed by primary sclerosing cholangitis (PSC, n = 4), re-cirrhosis after LTX (n = 2), autoimmune hepatitis (AIH; n = 1), porphyria (n = 1), chronic hepatitis B (n = 1) and secondary sclerosing cholangitis (n = 1). At the time of ICU admission, most patients presented with CPS C (n = 16), while 5 patients showed CPS B. The median CPS score was 12 (IQR: 10–14). Infections (n = 12) and gastrointestinal bleeding events (n = 8) were the main precipitating events for the development of ACLF. The median number of organ failures at ICU admission was 4 (IQR: 4–6), corresponding to a median CLIF-C ACLF and CLIF-C OF Score of 67 (IQR: 57–76) and 15 (IQR: 14–18), respectively. We reported a median ICU length of stay (LOS) of 8 days (IQR: 3–13) and an ICU survival rate of 23.8% (n = 5). Additionally, we detected a 1-month survival of 23.8% (n = 5) and a 3-month survival of 19% (n = 4). Based on the CLIF-C-ACLF score, a predicted 1-month mortality of 82% and a predicted 3-month mortality of 92.9% were determined. Detailed information on the individual patients’ courses during CytoSorb adsorber application is shown in Fig. 1 and Supplemental Figure S2. The temporal relationship between CVVHD, CytoSorb application, outcome, and anticoagulation is depicted in Supplemental Table S1.

**Table 1:** Comparison of baseline characteristics between ICU Survivors and Non-Survivors.

	All patients	Survivors	Non-survivors
N (%)	21 (100)	5 (23.8)	16 (76.2)
Age, median (IQR)	50 (35–58)	47 (35–62)	51 (38.8–58)
male/female	18/3	5/0	13/3
Child–Pugh-Score, median (IQR)	12 (10–14)	12 (9–13)	13 (10–14)
Etiology of Cirrhosis, n (%)			
ALD	11 (52.4)	2 (40)	9 (56.3)
PSC	4 (19)	2 (40)	2 (12.5)
SSC	1 (4.8)	1 (20)	0
Chronic Hep. B	1 (4.8)	0	1 (6.3)
AIH	1 (4.8)	0	1 (6.3)
RC	2 (9.5)	0	2 (12.5)
Porphyria	1 (4.8)	0	1 (6.3)
Precipitating event for ACLF, n (%)			
Bleeding	8 (38.1)	1 (20)	7 (43.8)
Infection	12 (57.1)	3 (60)	9 (56.3)
Others	1 (4.8)	1 (20)	0
ICU LOS (days), median (IQR)	8 (3–13)	9 (5–25.5)	5.5 (3–12.5)
Vasopressor therapy, n (%)	20 (95.2)	4 (80)	16 (100)
Vasopressor resolved after CS, n (%)	6 (28.6)	5 (100)	1 (6.3)
MIV, n (%)	18 (85.7)	4 (80)	14 (87.5)
Length of MIV (days), median (IQR)	4 (2–8)	2 (1–9)	4 (2–8.5)
CS Adsorbers, median (IQR)	4 (2.5–8)	8 (6–10.5)	3 (2–4)
Duration of CS in hours, median (IQR)	64 (42.5–130)	156 (62.5–209.5)	59.5 (35–80.5)
SAPSII^a^, median (IQR)	59 (53–69)	68 (54–69)	59 (53–68)
SOFA^a^, median (IQR)	16 (13–19)	16 (15–17)	16.5 (11.8–19)
Number of OF^b^, median (IQR)	4 (4–6)	4 (3–4)	5 (4–6)
CLIF-C ACLF Score^b^, median (IQR)	67 (57–76)	60 (47–66)	68.5 (59.3–76.5)
CLIF-C OF Score^b^, median (IQR)	15 (14–18)	14 (13–14)	16.5 (15–18)

*ACLF* acute on chronic liver failure, *AIH* Autoimmune Hepatitis, *ALD* alcoholic liver disease, *CS* CytoSorb adsorber therapy, *f* female, *Hep B* hepatitis B, *ICU LOS* Intensive Care Unit Length of Stay, *LTX* liver transplantation, *m* male, *MIV* mechanical invasive ventilation, *n* population size, *OF* organ failures, *PSC* primary sclerosing cholangitis, *RC*, Re-cirrhosis after LTX, *SAPSII* simplified acute physiology score II, *SOFA* sequential organ failure assessment score, *SSC* secondary sclerosing cholangitis.

^a^Child–Pugh-, SAPSII and SOFA score were calculated within the first 24 h after admission.

^b^Organ Failures, CLIF-C ACLF Score and CLIF-C OF Score were calculated directly prior to CytoSorb therapy.

**Table 2:** Patients’ characteristics.

Pt	Age	m/f	Etiology of cirrhosis	CPS	ACLF trigger	Cerebral failure^a^	SAPSII score^b^	SOFA score^b^	MIV^c^	VP^c^	VP res. after CS	ACLF Grade	Nr. of OF	CLIF-C OF score^d^	CLIF-C ACLF score^d^	ICU survival	1-month survival	3-months survival	Reason for death
1	40	m	Porphyria	B8	Bleeding	0	53	11	Yes	Yes	No	3	4	15	50	No	No	No	MOF
2	58	m	ALD	C15	Infection	IV	71	20	Yes	Yes	No	3	5	17	80	No	No	No	MOF
3	61	m	ALD	C11	Bleeding	0	73	17	Yes	Yes	No	3	3	13	68	No	No	No	HS
4	48	m	PSC	B9	Infection	II	55	11	Yes	Yes	No	3	4	15	67	No	No	No	HS
5	47	m	ALD	C13	Bleeding	I	75	16	Yes	Yes	Yes	3	4	16	69	Yes	Yes	Yes	x
6	35	m	PSC	C14	Bleeding	III	53	19	Yes	Yes	No	3	6	18	67	No	No	No	MOF
7	25	f	Hep B	C13	Bleeding	IV	53	17	Yes	Yes	No	3	6	18	69	No	No	No	MOF
8	62	m	SSC	C12	Infection	0	68	17	Yes	Yes	Yes	3	4	14	66	Yes	Yes	Yes	x
9	27	f	AIH	C14	Infection	IV	27	9	Yes	Yes	No	3	6	18	60	No	No	No	MOF
10	65	m	ALD	C10	Infection	I	72	13	No	Yes	No	3	3	15	74	No	No	No	septic shock
11	52	m	ALD	C14	Infection	III	66	20	Yes	Yes	No	3	6	18	76	No	No	No	HS
12	43	m	ALD	C14	Infection	I	59	19	Yes	Yes	No	3	5	17	80	No	No	No	MOF
13	25	f	RC	B9	Bleeding	0	59	12	Yes	Yes	No	3	5	16	48	No	No	No	MOF
14	32	m	ALD	C13	Infection	0	54	19	Yes	Yes	Yes	3	4	14	60	Yes	Yes	Yes	x
15	54	m	ALD	C11	Infection	0	61	14	Yes	Yes	No	3	4	14	48	No	No	No	MOF
16	62	m	PSC	B9	MCI	0	69	15	No	No	Yes	2	2	10	47	Yes	Yes	No	MOF
17	73	m	ALD	C10	Bleeding	0	43	16	Yes	Yes	No	3	4	15	76	No	No	No	MOF
18	58	m	ALD	C15	Infection	IV	67	19	Yes	Yes	Yes	3	6	18	82	No	No	No	MOF
19	35	m	PSC	B9	Infection	I	45	15	Yes	Yes	Yes	3	3	13	45	Yes	Yes	Yes	x
20	50	m	ALD	C15	Bleeding	III	75	21	Yes	Yes	No	3	6	18	78	No	No	No	MOF
21	54	m	RC	C12	Infection	II	44	11	No	Yes	No	3	3	13	57	No	No	No	MOF

*AIH* autoimmune hepatitis, *ALD* alcoholic liver disease, *ALF* acute liver failure; adverse effects including CytoSorb associated bleedings or other complications, *CPS* Child–Pugh Score, *f* female, *HS* hemorrhagic shock, *LTX-R* Liver transplantation rejection, *MCI* myocardial infarction, *MIV* mechanical invasive ventilation, *MOF* multi organ failure, *m* male, *Nr. of OF* Number of organ failures, *PSC* primary sclerosing cholangitis, *Pt*. patient, *RC* Re-cirrhosis, *SAPSII* simplified acute physiology score II, *SOFA* sequential organ failure assessment score, *SSC* secondary sclerosing cholangitis, *VP* vasopressor, *VP res. after CS* Vasopressor resolved after CytoSorb.

^a^According to West Haven criteria.

^b^SAPSII and SOFA score were calculated within the first 24 h after admission.

^c^MIV and Vasopressor therapy during ICU stay.

^d^Child–Pugh Score, CLIF-C ACLF Score and CLIF-C OF Score were calculated directly prior to CytoSorb therapy.

All but one patient (95.2%) required vasopressor (i.e., noradrenaline) therapy during ICU stay. A detailed description of noradrenaline doses during CytoSorb treatment is depicted in Supplemental Table S2. In 10 patients, an increase in noradrenalin dose was observed 6 h after the start of CytoSorb treatment. After discontinuation of CytoSorb therapy, 6 patients (28.6%) were resolved from vasopressors. In addition, mechanical invasive ventilation (MIV) was conducted in 18 patients with a median length of MIV of 4 days (IQR: 2–8).

Differences in baseline characteristics between survivors and non-survivors are shown in Table 1. Survivors were younger (47 vs. 51 years), showed a lower CLIF-C-ACLF score at ICU admission (60 vs. 68.5), and had a longer ICU LOS (9 vs. 5.5 days).

### Changes in laboratory parameters

**Figure 2:**
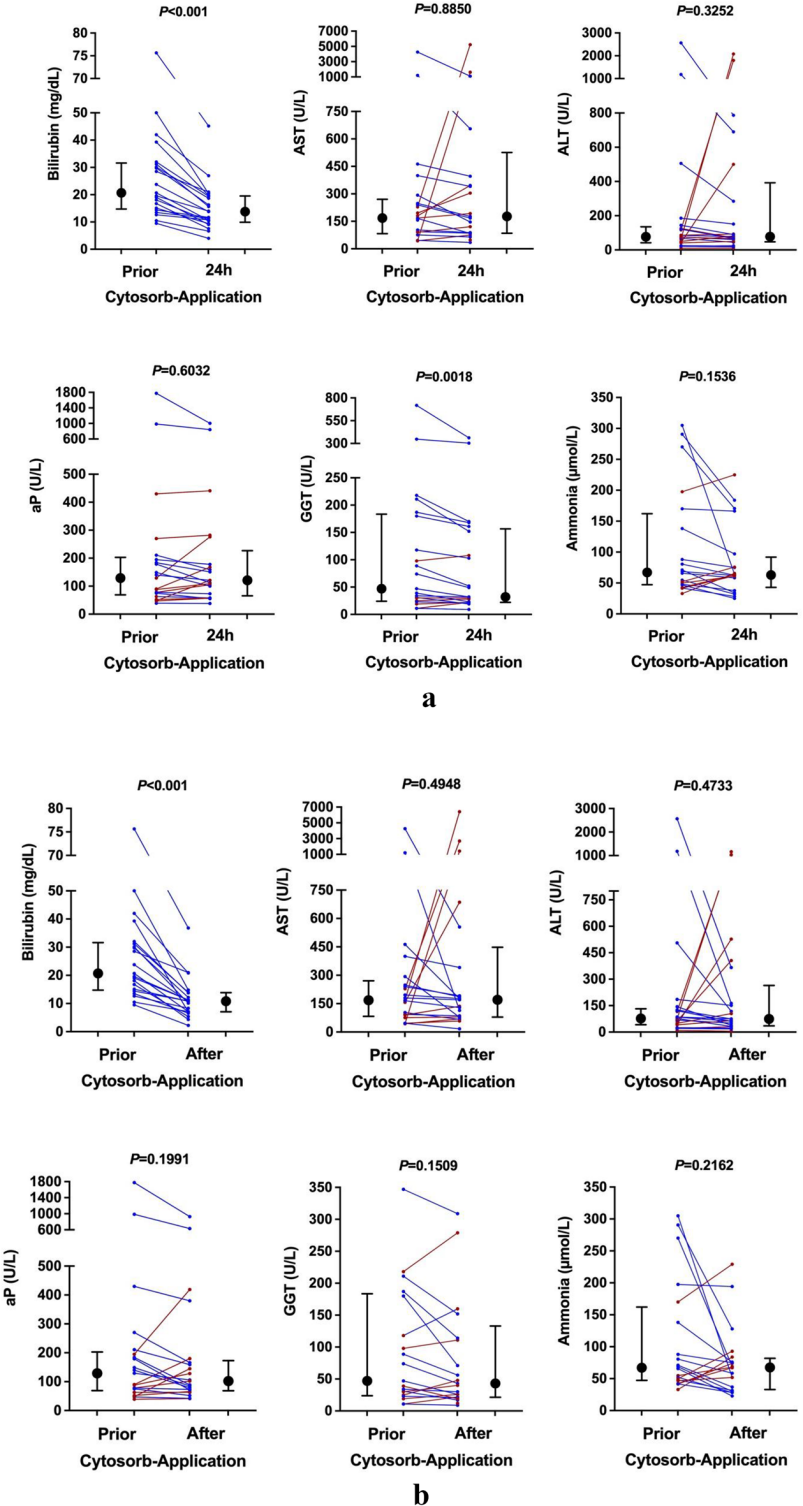
Evolution of liver chemistries in ACLF patients prior to and after CytoSorb adsorber application. Black dots and error bars indicate the median and interquartile range of bilirubin, AST, ALT, aP, GGT, and ammonia levels prior, 24 h after the commencement of CytoSorb adsorber therapy (**a**), and after CytoSorb adsorber therapy (**b**). The red and blue dots represent the individual patients' values prior to and 24 h after the commencement of CytoSorb adsorber therapy (**a**) and after CytoSorb adsorber therapy (**b**) from left to right. Patients with decreased or unchanged liver chemistries are shown in blue, while those with increased liver chemistries are shown in red. Data of ammonia was missing in one patient. *aP* alkalic phosphatase, *ALT* alanine aminotransferase, *AST* aspartate transferase, *GGT* gamma-glutamyl transferase, *mg/dl* milligram per deciliter, *U/L* units per liter, *µmol/l* micromole per liter.

The median number of CytoSorb applications per patient was 4 (IQR: 2.5–8), with a median CytoSorb therapy duration of 64 h (IQR: 42.5–130). We found a significant decrease in bilirubin levels during and after CytoSorb therapy (median peak: 20.7 mg/dL to median post-treatment: 10.8 mg/dL; – 47.8%; *p* < 0.001; Fig. 2). Interestingly, these changes were already observed after 24 h of CytoSorb treatment (median peak: 20.7 mg/dL to median 24 h-treatment: 13.8 mg/dL; – 33.3%; *p* < 0.001; Fig. 2a). Except for a significant decrease in GGT levels after 24 h (median peak: 47 U/L to median post 24 h-treatment: 32 U/L; – 31.9%; *p* = 0.0018), we did not find a clear trend for the remaining liver chemistries.

**Figure 3:**
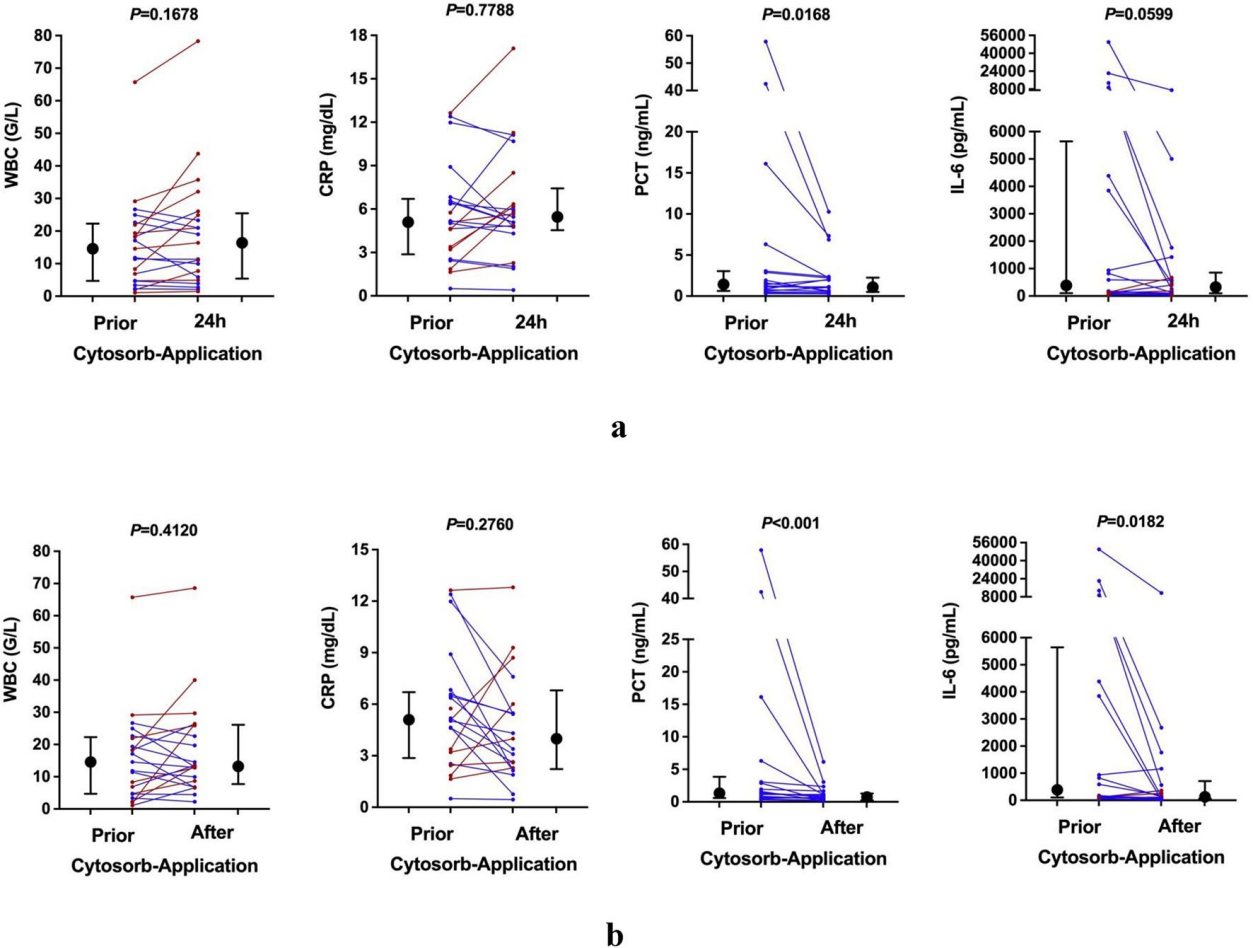
Evolution of inflammatory parameters in ACLF patients before and after CytoSorb adsorber application. Black dots and error bars indicate the median and interquartile range of CRP, PCT, IL-6, and leukocyte levels prior to and 24 h after the commencement of CytoSorb adsorber therapy (**a**) and after total CytoSorb adsorber therapy (**b**). The red and blue dots represent the individual patients' values prior to and 24 h after the commencement of CytoSorb adsorber therapy (**a**) and after total CytoSorb adsorber therapy (**b**) from left to right. Patients with decreased or unchanged inflammatory parameters are shown in blue, while patients with increased inflammatory parameters are shown in red. Data of PCT and IL-6 was missing in 3 patients. *CRP* C-reactive protein, *IL-6* interleukin 6, *mg/dl* milligram per deciliter, *ng/mL* nanogram per milliliter, *pg/mL* picogram per milliliter, *PCT* procalcitonin.

WBC, CRP, PCT, and IL-6 were monitored as surrogate parameters for systemic inflammation. While CytoSorb therapy did not affect WBC and CRP levels, we observed a significant decline in PCT levels (median peak: 1.34 ng/mL to median 24 h-treatment: 1.09 ng/mL; – 18.4%; *p* < 0.001) 24 h after first CytoSorb adsorber therapy (Fig. 3a). There was also a relevant but non-significant trend regarding the decline in IL-6 levels after 24 h of CytoSorb therapy (median peak: 385 pg/mL to median 24 h-treatment: 327 pg/mL; – 15.2%; *p* = 0.0599). Importantly, after total CytoSorb therapy, both PCT (median peak: 1.34 ng/mL to median post-treatment: 0.74 ng/mL; – 44.9%; *p* < 0.001) and IL-6 (median peak: 385 pg/mL to median post-treatment: 131 ng/mL; – 66.1%; *p* = 0.0182) showed a significant decrease (Fig. 3b).

In addition, the 5 ICU survivors tended to have a stronger initial decrease in bilirubin (– 76.1% vs. – 48.2%), procalcitonin (– 90.6% vs. – 23.5%), and IL-6 (– 54.6.3% vs. – 17.8%) upon CytoSorb treatment compared to non-survivors.

In order to analyze if the aforementioned changes in laboratory parameters were achieved by the addition of CytoSorb adsorber to CVVHD and not by conventional hemodialysis alone, we also evaluated laboratory changes in patients with ACLF and CVVHD without inclusion of the CytoSorb adsorber. The basic characteristics of this control group are depicted in Supplemental Table S3–S4. As seen in Supplemental Table S5, CVVHD without CytoSorb had no significant impact on the investigated laboratory parameters. In the control group, we detected a noticeable but not statistically significant decrease in median ammonia levels after 24 h and at CVVHD discontinuation.

### Feasibility, safety, and complications of CytoSorb therapy in ACLF patients

**Figure 4:**
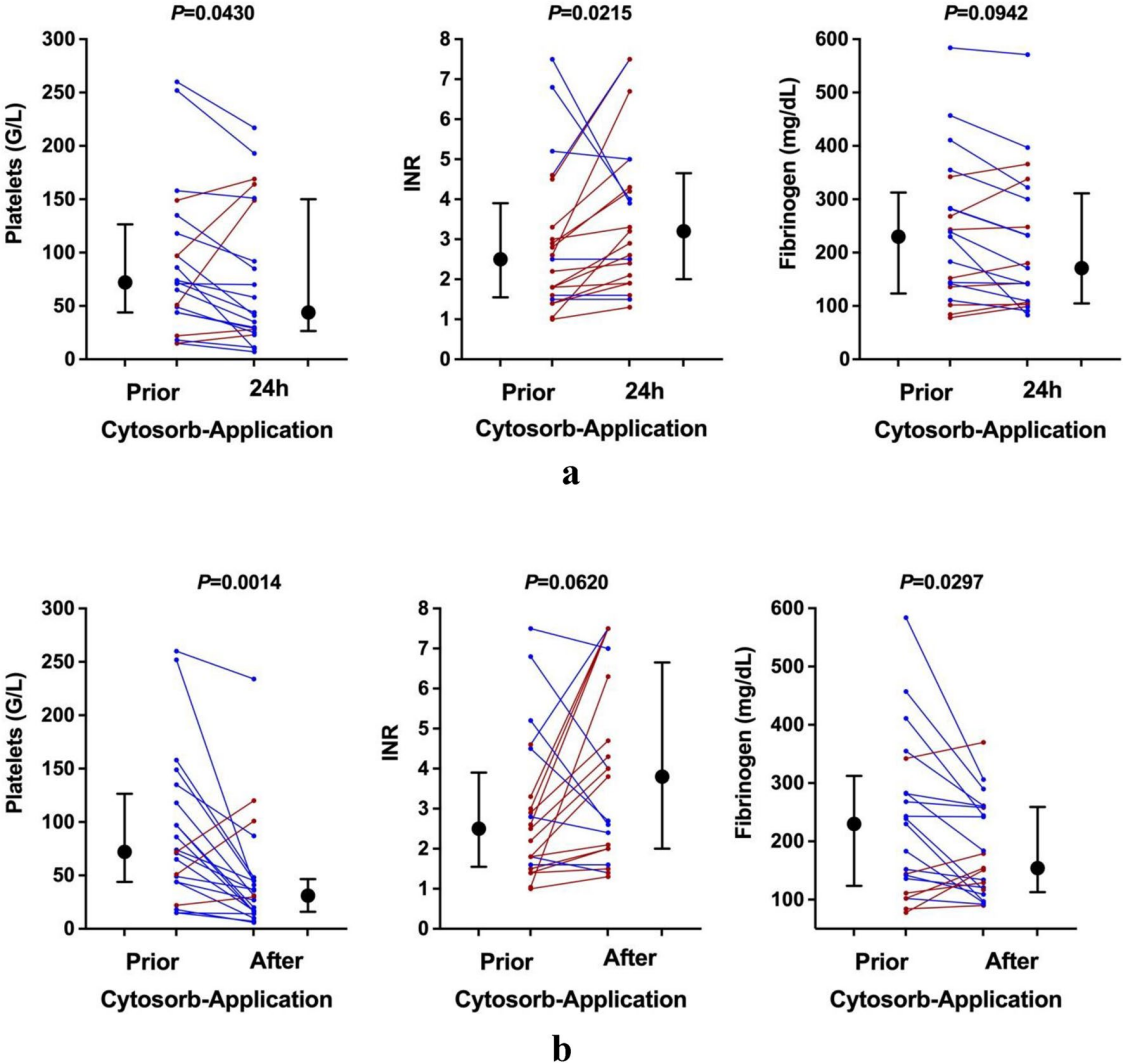
Evolution of coagulation parameters in ACLF patients prior to and after CytoSorb adsorber application. Black dots and error bars indicate the median and interquartile range of platelet count, INR, and fibrinogen levels prior, 24 h after the commencement of CytoSorb adsorber therapy (**a**) and after total CytoSorb adsorber therapy (**b**). The red and blue dots represent the individual patients' values prior to and 24 h after the commencement of CytoSorb adsorber therapy (**a**) and after total CytoSorb adsorber therapy (**b**) from left to right. Patients with a decrease in or unchanged platelet count are shown in blue, while patients whose platelet count remained unchanged or increased are shown in red. *G/L* Giga per liter, *INR* international normalized ratio, *mg/dl* milligram per deciliter.

We found a significant decrease in platelet counts during and after CytoSorb therapy (median peak: 72 G/L to median post-treatment: 31 G/L; – 56.9%; *p* = 0.0014; Fig. 4). These changes could already be detected 24 h after the start of CytoSorb treatment (median pre-treatment: 72 G/L to median 24 h-treatment: 44 G/L; – 38.9%; *p* = 0.043). Concerning global coagulation tests, we observed a significant increase in INR after 24 h of CytoSorb therapy (median pre-treatment: 2.5 to median 24-treatment: 3.2; 28%; *p* = 0.0215). In addition, fibrinogen levels showed a significant decrease during the course of CytoSorb therapy (median pre-treatment: 230 mg/dL to median post-treatment: 154 mg/dL; 33%; *p* = 0.0297). We did not observe any bleeding or other complications (i.e., allergic reactions) attributed to treatment with the CytoSorb adsorber in our ACLF patient cohort.

In all patients, the CytoSorb adsorber was combined with CVVHD using regional citrate anticoagulation by local standards. In our ACLF cohort, citrate accumulation was suspected in 6 (28.6%) patients, and in all of these cases, citrate anticoagulation was replaced by substituting antithrombin III (Fig. 1 and Supplemental Table S1)^21^. In these patients, antithrombin III anticoagulation was continued for the remaining CVVHD circuits.

## Discussion

Here we provide first observational data on the use of the CytoSorb adsorber as supportive therapy in ACLF patients. Importantly, we detected a significant reduction of bilirubin after 24 h and at the end of CytoSorb application in all ACLF patients. In addition, CytoSorb therapy also led to a significant decrease in PCT and IL-6 levels after CytoSorb application, indicating a potential benefit by ameliorating the systemic proinflammatory state in ACLF. We also found a significant decrease in platelets and alterations in the plasmatic coagulation, but did not detect bleeding and other complications directly attributed to CytoSorb use.

The integration of CytoSorb in an extracorporeal circuit to modulate hyperinflammation (‘cytokine storm’) in sepsis represents a novel therapeutic approach first introduced in 2008^29–31^. CytoSorb treatment has been approved in Europe since 2011 and is authorized for use as an additional treatment for all indications associated with elevated cytokine levels. In animal studies, CytoSorb treatment was able to reduce inflammation-related molecules such as IL-6, IL-10, and TNF- α^32,33^. *Brouwer *et al. demonstrated reduced 28-day mortality in septic patients treated with CytoSorb^31^. Supporting data for the use of CytoSorb in the setting of ACLF are derived from patients with ALF (i.e., notably a different setting) occurring in patients with sepsis and systemic inflammation. Importantly, systemic inflammation is a key risk factor for the development and progression of ACLF^6,8^. Therapy with CytoSorb adsorber in ACLF patients is used with the rationale to ameliorate a hyperinflammatory state and thereby promote liver regeneration^5,11,12^. While we did not find any clear trend regarding CRP and WBC during and after CytoSorb treatment, we detected a significant decline in PCT and IL-6 levels^13^. Importantly, the declines in PCT and IL-6 levels were already evident as early as 24 h after the start of CytoSorb application, i.e., in a critical early phase of ACLF. This finding argues for a direct effect of the CytoSorb adsorber on these proinflammatory parameters. However, in our study, the reported decrease in IL-6 was rather small compared to in vitro data^34^. An explanation for the discrepancy between in vitro experiments and clinical application might be the constant hyperinflammatory response during ACLF, especially at the beginning of the disease course. In this regard, the observed small decrease of IL-6 by CytoSorb therapy might indicate a successful dampening of hyperinflammation that would have otherwise happened. Similar effects could already be seen in a study investigating the effects of CytoSorb hemoperfusion on systemic inflammation in humans in vivo^35^.

We also found a significant decrease in bilirubin levels during hemoadsorption, confirming data from other studies analyzing the effects of CytoSorb in different settings^14,36–39^. Again, these effects could already be achieved as early as 24 h after the commencement of CytoSorb therapy. According to recent data, the impact of CytoSorb treatment on bilirubin removal is even more effective compared to MARS^13,40^. However, the removal of further clinically relevant metabolites accumulating during liver failure by the CytoSorb adsorber is still unclear and under debate. Effects on hyperammonemia have been reported mainly in ex vivo studies^40,41^. However, recent data from one clinical report indicate that CytoSorb does not directly affect ammonia levels, and the elimination may be mainly achieved through simultaneously applied hemodialysis^42^. Interestingly, we found a more pronounced decrease in median ammonia levels in the control group, which only received CVVHD. However, the sample size was small, and the results were not statistically significant. It is yet not clear how CytoSorb application influences ammonia levels in patients with ACLF. Recent data suggests that the decrease in ammonia levels is a primary effect of hemodialysis, which is a perquisition for CytoSorb use^43^. However, we do not think CytoSorb negatively affects serum ammonia levels. Therefore, the observed difference in the clearance of ammonia levels between the CytoSorb group and the control group is likely due to the small sample size in both subgroups. Further studies are needed to investigate the impact of CytoSorb therapy on hyperammonemia and encephalopathy during liver failure. Nevertheless, our findings suggest that hemoadsorption with CytoSorb might support blood detoxification during ACLF.

Besides standard medical treatment, there is considerable interest in extracorporeal liver support systems for patients with liver failure^15,44^. Artificial liver support devices, such as MARS, Prometheus, and single-pass albumin dialysis (SPAD), led to an amelioration of laboratory parameters in ACLF. However, no consistent improvement in survival was found compared to standard medical treatment^15,44^. A prospective, randomized controlled trial reported no benefit in laboratory chemistries and outcome in ACLF patients treated with ELAD^45^. Potential positive effects were reported in ALF patients with high volume plasma exchange, while a survival benefit in ACLF patients is debated controversially^15^.

There is a lack of data on the efficacy of CytoSorb adsorber in the specific setting of ACLF. In a recent retrospective study of a small patient cohort with liver failure (ALF and ACLF) by *Popescu *et al.^40^, CytoSorb therapy was found to be equally or even more effective in rebalancing liver functional tests in patients with liver failure compared to MARS. However, there were no differences in patient outcome^40^. Nevertheless, CytoSorb adsorber is a rather cheap, easy-to-use, and readily available blood purification tool installed in conventional hemodialysis, which could also be used in smaller centers, in contrast to artificial liver support devices, that often require significant expertise for their application^38^.

The application of CytoSorb adsorber is generally associated with a low risk of complications^46^. In our cohort of ACLF patients, platelets were significantly decreased, as reported in other studies using renal replacement therapy with CytoSorb adsorber^40,47^. Moreover, we detected changes in plasmatic coagulation, including a significant decrease in fibrinogen levels and an increase in INR after the commencement of CytoSorb that may, however, also be due to liver disease progression. We did not detect bleeding complications or other adverse effects directly associated with the use of CytoSorb adsorber therapy. In 10 patients CytoSorb application was accompanied by increased vasopressor requirements shortly after commencement of adsorber therapy. This observation is best explained by progressive multiorgan failure and refractory shock in ACLF grade 3 rather than a negative impact of CytoSorb adsorber on hemodynamics itself.

Due to an imbalance of coagulation and risk of bleeding, regional citrate dialysis was used in all our patients, and a switch to antithrombin III supplementation due to citrate accumulation was only rarely necessary. Nevertheless, citrate accumulation was detected in 6 patients. In our experience, the risk for citrate accumulation is highest during the first cycle of CVVHD when the patient is still in an unstable condition. In this situation, the already limited liver function is further compromised by circulatory/septic shock, resulting in the inability to metabolize citrate and ultimately leading to citrate accumulation^20^. In this regard, citrate accumulation might also represent a negative prognostic marker for outcome in ACLF patients^48^.

The ICU survival of our study population was rather low, with 23.8%. However, our patients were admitted to the ICU with advanced ACLF as indicated by high CLIF-C ACLF scores and CLIF-OF scores at ICU admission corresponding to a predicted 1-month and 3-month mortality of 82% and 92.9%, respectively. 95.2% of our patients had an ACLF grade 3 before CytoSorb application. In our small study population, we observed comparable numbers with a 1-month mortality of 76.2% and 3-month mortality of 81%. Similar outcome data concerning ACLF patients was reported in the CANONIC study (28-day = 76.7%; 90-day mortality = 79.1%)^1^. Overall, in patients with advanced ACLF stages, treatment with CytoSorb adsorber might be less effective as organ failures may no longer be reversible. Therefore, close monitoring of organ function and, consequently, the earlier application of CytoSorb adsorber in ACLF patients might represent a treatment strategy to prevent disease progression to multiorgan failure (i.e., bridge to recovery) or to enable liver transplantation^5,49,50^. Indeed, liver transplantation for ACLF patients has shown very promising results, with a 1-year probability of survival after liver transplantation of 81%^50^. Unfortunately, liver transplantation for ACLF patients is not established at our center. Therefore, none of our patients was even evaluated for eligibility for liver transplantation.

The present study has limitations: First, in our single center study, we only report the data of a rather small cohort, including 21 ACLF patients treated with CytoSorb adsorber. However, to our knowledge, this is the first study to date that investigates the effects of hemoadsorption with CytoSorb in the specific setting of ACLF—while others have included patients with ALF in other settings^15,40^. Second, due to the retrospective design, we had some missing laboratory values, especially for ammonia, but also for PCT and IL-6, which may therefore introduce a bias in the interpretation of the results. Third, we acknowledge that in addition to CytoSorb therapy, patients always received standard medical treatment, including hemodialysis, according to the EASL guidelines^19^. Therefore, it is unclear to what extent the laboratory changes may be allocated to hemoadsorption or standard medical treatment. However, we found significant changes in a couple of laboratory parameters 24 h after the commencement of CytoSorb treatment, suggesting a direct effect of hemoadsorption. Fourth, due to the small number of patients in the control group, we were not able to perform a propensity matched comparison. Future multicenter prospective trials are required to provide a more comprehensive comparison regarding the efficacy of CVVHD + CytoSorb therapy and CVVHD alone in patients with ACLF. Nevertheless, we here present a comprehensive summary of our single center experience using CytoSorb adsorber in ACLF patients.

## Conclusions

Our study supports the use of CytoSorb adsorber as a feasible and easy-to-use blood purification tool with few complications in patients with ACLF. CytoSorb treatment led to a significant decrease in important surrogate markers of systemic inflammation and supported blood detoxification by removing bilirubin in ACLF patients. Larger randomized controlled trials are warranted to further investigate the clinical value of CytoSorb therapy in ACLF patients.

### Supplementary Information

Supplementary Information.

## Data Availability

Data are available from the corresponding author upon reasonable request.

## Abbreviations

ACLF: Acute-on-chronic liver failure
ALD: Alcoholic liver disease
ALF: Acute liver failure
ALT: Alanine aminotransferase
aP: Alkaline phosphatase
AIH: Autoimmune hepatitis
AST: Aspartate transferase
CRP: C-reactive protein
CVVHD: Continuous venovenous hemodialysis
CPS: Child–Pugh-score
DAMP: Damage-associated molecular patterns
EASL: European Association for the Study of the Liver
GGT: Gamma-glutamyl transferase
ICU: Intensive Care Unit
IL-6: Interleukin-6
INR: International normalized ratio
IQR: Interquartile range
LOS: Length of stay
LTX: Liver transplantation
MCI: Myocardial infarction
MIV: Mechanical invasive ventilation
MOF: Multi organ failure
PAMP: Pathogen-associated molecular patterns
PCT: Procalcitonin
Pt.: Patient
PSC: Primary sclerosing cholangitis
RC: Re-cirrhosis
SAPS II: Simplified acute physiology score
SOFA: Sequential organ failure assessment score
VP: Vasopressor
WBC: White blood count
