# Supplementary material for: Use of the CytoSorb adsorber in patients with acute-on-chronic liver failure
Source: Sci Rep. 2024 May 17;14:11309. doi: 10.1038/s41598-024-61658-3 (PMC11101465; doi:10.1038/s41598-024-61658-3)
Supplement: Supplementary file 1 — Supplementary Information. [file 41598_2024_61658_MOESM1_ESM.pdf]

Supplementary Material

Use of the CytoSorb adsorber in patients with acute on chronic liver failure

Haselwanter Patrick[1], Scheiner Bernhard[1], Balcar Lorenz[1], Semmler Georg[1], Riedl-Wewalka Marlene[1], Schmid Monika[1], Reiberger Thomas[1], Zauner Christian[1], Schneeweiß-Gleixner Mathias[1]

[1] Department of Medicine III, Clinical Division of Gastroenterology and Hepatology, Intensive Care Unit 13H1, Medical University of Vienna, Austria

Supplemental Figure S1: Flow chart of the study population.

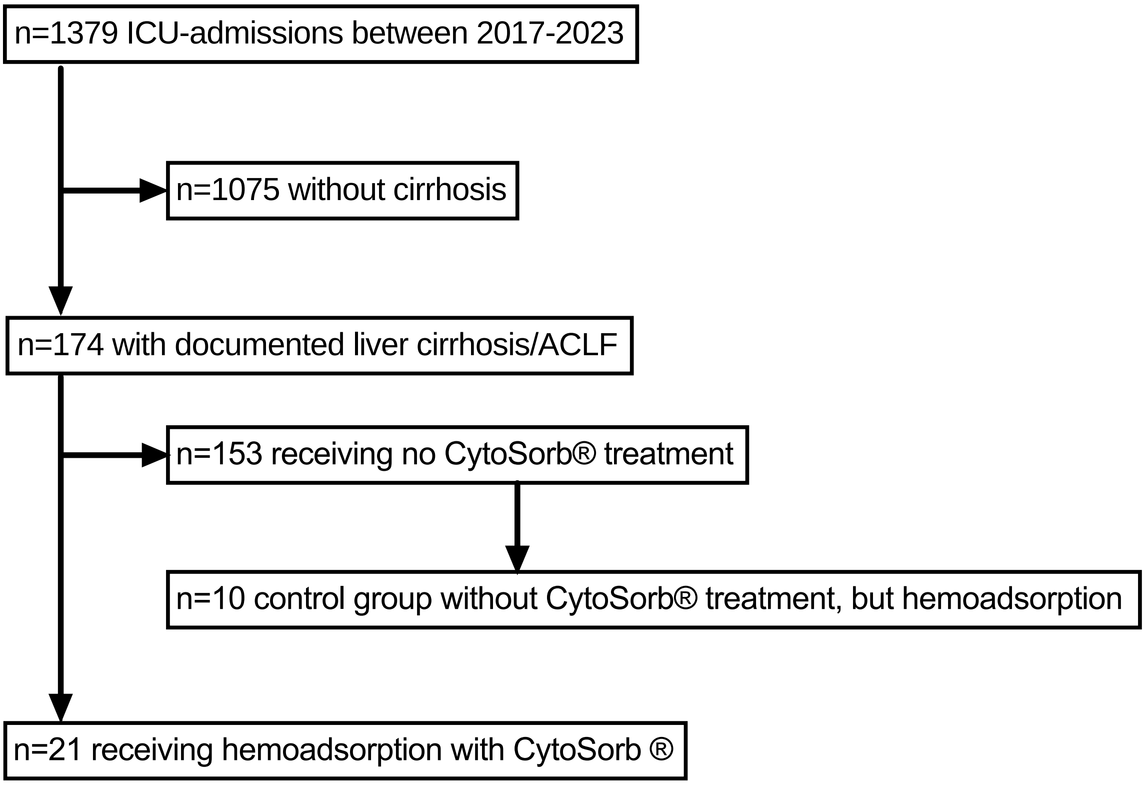

Haselwanter et al, Supplemental Figure S1

Supplemental Figure S2: Number of organ failures during CytoSorb therapy. The patient numbers refer to Table 2.

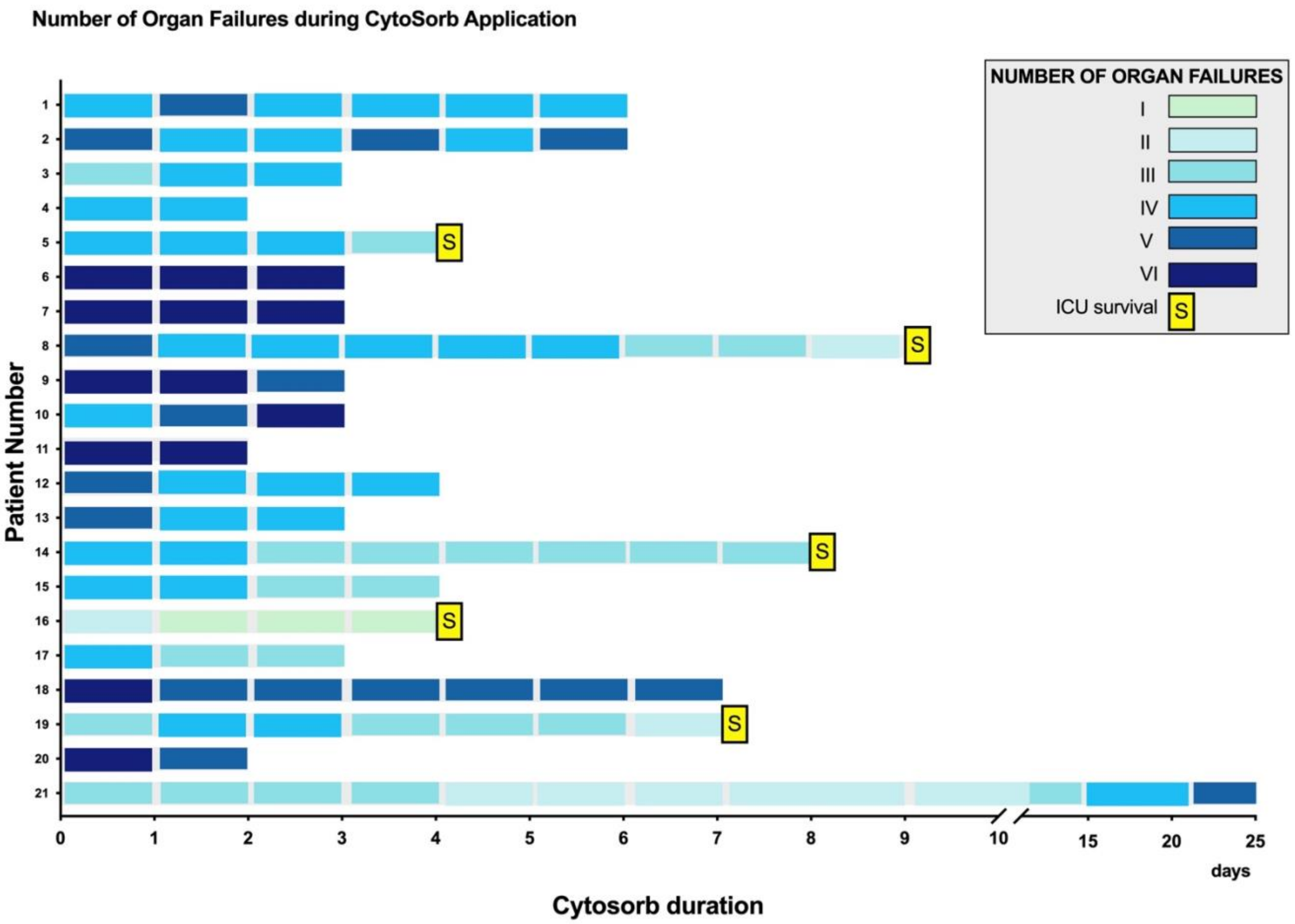

Haselwanter et al, Supplemental Figure S2

Supplemental Table S1: Temporal relationship between CVVHD, CytoSorb application, outcome and anticoagulation.

Pt.	CS duration (d)	CVVHD duration (d)	Last CS change to CVVHD end (d)	Last CS change to death date (d)	CVVHD end to death date (d)	CA	CVVHD start until AT-III (d)
**1**	6	6	0	0	0	yes	0
**2**	6	6	0	1	1	yes	0
**3**	3	12	7	12	5	no	x
**4**	2	2	0	0	0	no	x
**5**	4	8	4	x	x	no	x
**6**	3	3	0	1	1	yes	1
**7**	3	4	1	2	1	yes	2
**8**	9	10	1	x	x	no	x
**9**	3	4	1	1	0	yes	1
**10**	3	3	0	0	0	no	x
**11**	2	2	1	3	2	yes	0
**12**	4	4	0	0	0	no	x
**13**	3	10	6	7	1	no	x
**14**	8	10	1	x	x	no	x
**15**	4	9	5	8	3	no	x
**16**	4	4	0	81	81	no	x
**17**	3	4	1	1	0	no	x
**18**	7	7	1	8	7	no	x
**19**	7	32	25	x	x	no	x
**20**	2	2	0	1	1	no	x
**21**	25	25	0	0	0	no	x

In patients with the numbers 5, 8, 14 and 19, days between the last CytoSorb change and CVVHD discontinuation to death date were not applicable due to the survival in the observed period. Days between CVVHD start and AT-III application were only applicable in patients 1, 2, 6, 7, 9, and 11 due to citrate accumulation.

Abbreviations: AT-III, antithrombin III; CA; Citrate Accumulation CS, CytoSorb; CVVHD, continuous venovenous hemodiafiltration; d, days; Pt., patient; x, not applicable.

Supplemental Table S2: Noradrenaline doses (in µg/kg/min) during CytoSorb treatment.

	Noradrenaline doses (in µg/kg/min)			
**Pt.**	**At CytoSorb start**	**6 hours after CytoSorb start**	**At CytoSorb end**	**Peak dose**
**1**	0.463	0.752	0.208	1.9
**2**	0.13	0.296	0.278	0.37
**3**	1.5	1.33	0.031	1.5
**4**	0	0	0.427	0.427
**5**	0.108	0.074	0.025	0.108
**6**	0.286	0.381	0.179	0.893
**7**	0.182	0.364	0.427	0.455
**8**	0.041	0.071	0	0.386
**9**	0	0	0.3	0.3
**10**	0.896	1.03	0.986	1.075
**11**	0.102	0.285	0.183	0.427
**12**	0.396	0.396	1	1
**13**	0.212	0.04	0.034	0.212
**14**	0.236	0.222	0	0.4
**15**	0	0	0.163	0.46
**16**	0	0	0	0.254
**17**	0.324	0.196	0.176	0.324
**18**	0.01	0.088	0.039	0.294
**19**	0	0	0	0
**20**	0.036	0.079	0.122	0.122
**21**	0	0.042	0.506	0.633

Abbreviations: Pt., patient

Supplemental Table S3: Control group of ACLF patients receiving CVVHD without CytoSorb adsorber.

Pt.	Age	m/f	Etiology of cirrhosis	CPS	ACLF trigger	Cerebral failure*	SAPSII score⁺	SOFA score⁺	MIV$^{\times}$	VP$^{\times}$	VP res. after CS	ACLF Grade	Nr. of OF	CLIF-C OF score#	CLIF-C ACLF score#	ICU survival	1-month survival	3-months survival	Reason for death
**C1**	58	m	ALD	C11	CPR	0	43	20	yes	yes	no	2	2	11	51	no	no	no	MOF
**C2**	64	m	ALD	C11	bleeding	IV	55	13	no	no	yes	3	3	12	61	yes	yes	yes	x
**C3**	34	f	ALD	C11	bleeding	0	80	19	yes	yes	yes	2	2	12	48	no	no	no	MOF
**C4**	71	m	Hep B	C13	HE	IV	65	17	yes	yes	no	3	4	15	67	no	no	no	MOF
**C5**	59	f	ALD	C11	infection	IV	70	8	no	no	yes	2	2	11	61	no	no	no	MOF
**C6**	68	f	RC	B9	infection	I	59	12	no	no	yes	1	1	10	48	no	no	no	MOF
**C7**	37	f	AIH	C13	bleeding	IV	41	8	no	no	yes	3	4	16	49	yes	yes	yes	x
**C8**	70	m	ALD	B8	infection	0	53	9	yes	yes	yes	3	4	14	68	no	no	no	septic shock
**C9**	55	m	ALD	C11	bleeding	II	46	15	yes	yes	no	3	4	15	67	no	no	no	MOF
**C10**	44	m	ALD	C13	infection	0	22	8	yes	yes	yes	3	4	14	59	no	no	no	MOF

Abbreviations: ACLF, acute on chronic liver failure; AIH, autoimmune hepatitis; ALD, alcoholic liver disease; C1-C10, patient 1 to 10 in the control group; CPR, cardiopulmonary resuscitation; CPS, Child-Pugh Score; CS, CytoSorb; CVVHD, continuous venovenous hemodiafiltration; HE, hepatic encephalopathy; Hep B, hepatitis B; MIV, mechanical invasive ventilation; MOF, multiorgan failure; Nr. of OF, Number of organ failures; Pt., patient; RC, Re-cirrhosis; SAPS II, simplified acute physiology score II; SOFA, sequential organ failure assessment score; VP, vasopressor; VP res. after CS, Vasopressor resolved after CytoSorb; m, male; f, female

** According to West Haven criteria.*

⁺ SAPS II and SOFA scores were calculated within the first 24 hours after admission.

$^{\times}$ MIV and Vasopressor therapy during ICU stay

# Child-Pugh Score, CLIF-C ACLF Score, and CLIF-C OF Score were calculated directly before CVVHD

Supplemental Table S4: Comparison of baseline characteristics between patients with CVVHD + CytoSorb adsorber and patients in the control group with CVVHD.

	**Patients with CytoSorb**	**Patients without CytoSorb**
**N (%)**	21 (100)	10 (10)
**Age, median (IQR)**	50 (35-58)	58.5 (46.8-67)
**male/female**	18/3	6/4
**Child-Pugh-Score, median (IQR)**	12 (10-14)	11 (11-13)
**Etiology of Cirrhosis, n (%)**		
ALD	11 (52.4)	7 (70)
PSC	4 (19)	0
SSC	1 (4.8)	0
Chronic Hep. B	1 (4.8)	1 (10)
AIH	1 (4.8)	1 (10)
RC	2 (9.5)	1 (10)
Porphyria	1 (4.8)	0
**Precipitating event for ACLF, n (%)**		
Bleeding	8 (38.1)	4 (40)
Infection	12 (57.1)	4 (40)
Others	1 (4.8)	2 (20)
**ICU LOS, median (IQR)**	8 (3-13)	21 (6.3-35)
**Vasopressor therapy, n (%)**	20 (95.2)	6 (60)
**Vasopressor resolved after CS, n (%)**	6 (28.6)	7 (70)
**MIV, n (%)**	18 (85.7)	6 (60)
**Length of MIV (days), median (IQR)**	4 (2-8)	10 (3.3-22)
**CS Adsorbers, median (IQR)**	4 (2.5-8)	x
**Duration of CS in hours, median (IQR)**	64 (42.5-130)	x
**SAPSII*, median (IQR)**	59 (53-69)	54 (43.8-63.5)
**SOFA*, median (IQR)**	16 (13-19)	12.5 (8.3-16.5)
**Number of OF**, median (IQR)**	4 (4-6)	3.5 (2-4)
**CLIF-C ACLF Score**, median (IQR)**	67 (57-76)	60 (49.5-65.5)
**CLIF-C OF Score**, median (IQR)**	15 (14-18)	13 (11.3-14.8)

Abbreviations: ACLF, acute on chronic liver failure; AIH, Autoimmune Hepatitis; ALD, alcoholic liver disease; CS, CytoSorb adsorber therapy; CLIF, chronic liver failure; f, female; Hep B, hepatitis B; ICU LOS, Intensive Care Unit Length of Stay; LTX, liver transplantation; m, male; MIV, mechanical invasive ventilation; n, population size; OF, organ failures; PSC, primary sclerosing cholangitis; RC, Re-cirrhosis after LTX; SAPSII, simplified acute physiology score II; SOFA, sequential organ failure assessment score; SSC, secondary sclerosing cholangitis; x, not applicable.

** Child-Pugh-, SAPSII and SOFA score were calculated within the first 24 hours after admission.*
*** Organ Failures, CLIF-C ACLF Score and CLIF-C OF Score were calculated directly prior to CytoSorb therapy or CVVHD therapy.*

Supplemental Table S5: Laboratory changes in patients with CytoSorb vs. control group.

Laboratory parameters	CytoSorb group	p-value	control group	p-value
**Bilirubin n (%)**	21 (100)		10 (100)	
decrease after 24 hours in %	**33.33**	**<0.0001**	1.08	0.2012
decrease at the end in %	**47.75**	**<0.0001**	-34.09	0.1934
**ASAT n (%)**	21 (100)		10 (100)	
decrease after 24 hours in %	-5.36	0.8850	-8.54	0.3105
decrease at the end in %	-1.19	0.4948	11.85	0.4316
**ALAT n (%)**	21 (100)		10 (100)	
decrease after 24 hours in %	-1.30	0.3252	8.12	0.9434
decrease at the end in %	2.60	0.4733	46.58	0.2754
**GGT n (%)**	21 (100)		10 (100)	
decrease after 24 hours in %	**31.91**	**0.0018**	-3.33	0.4453
decrease at the end in %	8.51	0.1509	-120	0.8262
**AP n (%)**	21 (100)		10 (100)	
decrease after 24 hours in %	6.20	0.6032	-7.18	0.8223
decrease at the end in %	20.93	0.1991	-3.87	0.4922
**Ammonia n (%)**	20 (95.23)		8 (80)	
decrease after 24 hours in %	6.25	0.1536	48.87	0.1484
decrease at the end in %	-0.67	0.2162	58.82	0.5469
**PCT n (%)**	18 (85.71)		x	
decrease after 24 hours in %	**18.35**	**0.0168**	x	x
decrease at the end in %	**44.94**	**0.0002**	x	x
**IL-6 n (%)**	18 (85.71)		1 (10)	
decrease after 24 hours in %	15.17	0.0599	71.92	x
decrease at the end in %	**66.07**	**0.0182**	82.07	x
**CRP n (%)**	21 (100)		10 (100)	
decrease after 24 hours in %	-7.06	0.7788	-16.21	0.9219
decrease at the end in %	21.76	0.2760	11.28	0.4316
**WBC n (%)**	21 (100)		10 (100)	
decrease after 24 hours in %	-12.64	0.1678	-12.12	0.3223
decrease at the end in %%	9.20	0.4120	7.37	0.9219
**Platelets n (%)**	21 (100)		10 (100)	
decrease after 24 hours in %	**38.89**	**0.0430**	37.91	0.2207
decrease at the end in %	**56.94**	**0.0014**	**58.17**	**0.0449**
**Fibrinogen n (%)**	21 (100)		10 (100)	
decrease after 24 hours in %	25.65	0.0942	9.38	0.1016
decrease at the end in %	**33.04**	**0.0297**	21.88	0.4316
**INR n (%)**	21 (100)		10 (100)	
decrease after 24 hours in %	**-28.00**	**0.0215**	5	0.5078
decrease at the end in %	-52.00	0.0620	-12.5	0.2910
**Lactate n (%)**	21 (100)		10 (100)	
decrease after 24 hours in %	13.89	0.0621	-2.63	0.9922
decrease at the end in %	19.44	0.1936	15.79	0.2891

Changes in laboratory parameters are given as a relative decrease 24 hours after CytoSorb start and at the end of CytoSorb therapy. In the control group, laboratory changes are given as a relative decrease 24 hours after CVVHD start and at the end of CVVHD therapy. Positive values implicate a decrease in the median laboratory values, whereas negative values implicate an increase in laboratory parameters. Significant changes in laboratory parameters have been highlighted with bold format. In the control group, p-values of interleukin 6 and procalcitonin were not applicable due to a lack of recorded data. Values of procalcitonin in the control group were not available due to the retrospective study design.

Abbreviations: ASAT, aspartate aminotransferase; ALAT, alanine aminotransferase; AP, alkaline phosphatase; CRP, C-reactive protein; GGT, gamma-glutamyl transferase; IL-6, interleukin 6; INR, international normalized ratio; PCT, procalcitonin; WBC, white blood count; x, not applicable or not available.
